# Standardized ileal digestible amino acids and digestible energy contents in two modified soy protein concentrates and soybean meal fed to growing pigs^1^

**DOI:** 10.1093/tas/txac088

**Published:** 2022-06-26

**Authors:** Lee-Anne Huber, Cuilan Zhu, Lauren Hansen, Cierra Kozole, Cristhiam J Munoz Alfonso, Jessica Mark, Reza Akbari Moghaddam Kakhki, Youngji Rho, Elijah Kiarie

**Affiliations:** Department of Animal Biosciences, University of Guelph, Guelph, ON N1G 2W1, Canada; Department of Animal Biosciences, University of Guelph, Guelph, ON N1G 2W1, Canada; Department of Animal Biosciences, University of Guelph, Guelph, ON N1G 2W1, Canada; Department of Animal Biosciences, University of Guelph, Guelph, ON N1G 2W1, Canada; Department of Animal Biosciences, University of Guelph, Guelph, ON N1G 2W1, Canada; Department of Animal Biosciences, University of Guelph, Guelph, ON N1G 2W1, Canada; Department of Animal Biosciences, University of Guelph, Guelph, ON N1G 2W1, Canada; Department of Animal Biosciences, University of Guelph, Guelph, ON N1G 2W1, Canada; Department of Animal Biosciences, University of Guelph, Guelph, ON N1G 2W1, Canada

**Keywords:** digestible energy, modified soy protein concentrates, pig, standardized ileal digestible amino acids

## Abstract

Six ileal-cannulated barrows (28.0 ± 1.3 kg initial BW) were used in a replicated 3 × 3 Latin square design with one additional period (*n* = 7 or 6) to determine standardized ileal digestible (SID) AA and digestible energy of two modified soy protein concentrates [MSPC1 and MSPC2] and soybean meal (SBM). Pigs were fed one of three cornstarch-based diets with either MSPC1 or MSPC2 or SBM as the sole source of AA at a rate of 2.8 times the estimated maintenance energy requirement. In each period, pigs were adapted to diets for 7 d followed by 2 d of fecal collection and subsequently, 2 d of continuous ileal digesta collection for 8 h. The SID of AA was calculated using basal endogenous losses from a previous study for pigs fed a nitrogen-free diet. The digestible energy of the ingredients was calculated according to the difference method using a nitrogen-free diet that contained the same cornstarch:sucrose:oil ratio as the three test diets. The total Lys content was 33% and 38% greater for MSPC1 vs. MSPC2 and SBM, respectively. The SID of crude protein was greater for MSPC1 (96.9%) than for SBM (91.3%; *P* < 0.05), whereas an intermediate value was observed for MSPC2 (94.3% ± 1.2%). The SID of Ile (93.8%), Leu (93.6%), Lys (93.9%), Phe (96.7%), and Val (93.2%) were not different between MSPC1 and MSPC2 but greater than for SBM (88.8% ± 1.3%, 87.8% ± 1.2%, 84.5% ± 1.7%, 92.9% ± 1.0%, 86.5% ± 1.7% for Ile, Leu, Lys, Phe, and Val, respectively; *P* < 0.05). The SID of His and Thr was greater for MSPC1 than MSPC2 and SBM (*P* < 0.05), which were not different. The SID of Met was greater for MSPC1 and SBM vs. MSPC2 (*P* < 0.05). The SID of Arg was greater for MSPC1 than MSPC2 and SBM (*P* < 0.05), and greater for MSPC2 than SBM (*P* < 0.05). The digestible energy was greater for MSPC1 (4,677 kcal/kg) than MSPC2 and SBM (average; 3,896 ± 239 kcal/kg; *P* < 0.05), which were not different. Therefore, the MSPC1 was a better source of SID Lys and digestible energy than either MSPC2 or SBM and could be used as a highly digestible protein ingredient in swine rations.

## INTRODUCTION

Soybean products are commonly used as protein sources in swine diets globally (Stein et al., 2008; Goerke et al., 2012). However, factors including residual trypsin inhibitors, variation in processing (i.e., oil extraction and heating), and allergenic proteins (glycinin and β-conglycinin) among others antinutritional factors reduce the nutritional value of soybean products for pigs (e.g., Li et al., 1991; Chen et al., 2011; Kiarie et al., 2020). Novel soybean processing methods have the potential to remove or deactivate various antinutritional factors. Specifically, soy protein concentrate is produced via aqueous ethanol extraction at temperatures greater than 50 °C, which deactivates allergenic proteins and removes water-soluble carbohydrates (Sissons et al., 1982). The resulting product is high in crude protein (>65%) but has supported variable growth performance when used as a complete replacement for conventional soybean meal in monogastric diets (e.g., Sohn et al., 1994; Lenehan et al., 2007; Galkanda-Arachchige and Davis, 2020). Ultimately, based on protein efficiency ratios in broiler chickens, it was determined that commercially available soy protein concentrate products were still suboptimal and required further processing (Leske et al., 1995). Therefore, a new processing method was developed that included a pH reduction step and fractionation based on particle size, which increased crude protein solubility by 7%–10%, reduced the contents of fibrous components from 24% to 10%, and reduced antinutritional factors (e.g., phytate, phenolics, and saponins; Markedal et al., 2019; Kiarie et al., 2021) to generate novel, modified soy protein concentrates (MSPC). For accurate swine diet formulation, the digestibility of crude protein and AA must be determined for novel feed ingredients. Therefore, the objective of this study was to determine the standardized ileal digestibility (SID) of crude protein and AA and digestible energy of two MSPC and conventional soybean meal (SBM) fed to growing pigs.

## MATERIALS AND METHODS

Animal care and use protocols were approved by the University of Guelph Animal Care and Use Committee (AUP #4439). Pigs were cared for in accordance with the Canadian Council on Animal Care guidelines (CCAC, 2009).

### Ingredients, Diets, and Experimental Design

The two MSPC batches were obtained after pH reduction, drying, and sifting from two different lots (Triple A; Hornsyld, Denmark) and SBM from University of Guelph feed mill (Guelph, ON, Canada; Table 1). Three cornstarch-based diets were formulated to contain each test ingredient as the sole source of AA and to achieve crude protein content of approximately 20% (as-fed; Table 2). All other nutrients met or exceeded estimated requirements for growing pigs (NRC, 2012) and titanium dioxide was included to determine nutrient and energy digestibilities (e.g., de Lange et al., 1998).

**Table 1.** Analyzed nutrient composition (as-fed basis) of two modified soy protein concentrates and soybean meal

Item	MSPC1^1^	MSPC2^2^	Soybean meal^3^
Dry matter, %	93.27	92.17	89.54
Crude protein, %	69.06	62.67	47.21
Gross energy, kcal/kg	5,174	5,101	4,815
Calcium, %	0.14	0.17	0.18
Phosphorus, %	0.60	0.54	0.64
NDF, %^4^	14.40	13.84	8.78
Indispensable AA, %^5^			
Arg	4.76	4.92	3.88
His	1.68	2.00	1.46
Ile	3.19	3.19	2.23
Leu	5.62	5.23	3.89
Lys	4.47	3.01	2.75
Met	0.96	0.95	0.81
Phe	3.62	4.84	3.02
Thr	2.71	2.43	2.02
Val	3.17	3.05	2.20
Dispensable AA, %			
Ala	3.63	2.53	2.08
Asp	9.29	6.14	5.70
Cys	1.06	1.30	0.88
Glu	13.82	10.03	9.28
Gly	3.21	3.31	2.27
Pro	3.75	3.49	2.63
Ser	5.01	3.57	2.85
Tyr	2.22	2.84	1.81
Lys: crude protein	6.47	4.80	5.83
Mean particle size, µm	218	245	280
Mean particle size SD, µm	11	4	6

Modified soy protein concentrate 1 (Triple A, Hornsyld, Denmark).

Modified soy protein concentrate 2 (Triple A, Hornsyld, Denmark).

Soybean meal, dehulled, solvent extracted (Floradale Feed Mill Ltd., Floradale, ON, Canada).

NDF = neutral detergent fiber.

Trp was not analyzed.

**Table 2.** Ingredient composition (%, as-fed basis) of the test diets

Ingredient, %	MSPC1 diet^1^	MSPC2 diet^2^	SBM diet^3^
MSPC1	30.00	–	–
MSPC2	–	30.00	–
Soybean meal	–	–	43.36
Corn starch	58.75	58.75	47.52
Sucrose	4.27	4.27	3.46
Corn oil	1.43	1.43	1.15
Cellulose	1.43	1.43	1.15
Limestone	1.00	1.00	1.00
Monocalcium phosphate	1.80	1.80	1.00
NaCl	0.50	0.50	0.50
K_2_CO_3_	–	–	–
MgO	–	–	–
Vitamin and mineral premix^4^	0.60	0.60	0.60
TiO_2_	0.20	0.20	0.20
Calculated nutrient content, %^5^			
Crude protein	21.02	19.82	20.42
Calcium	0.88	0.88	0.83
Phosphorus	0.61	0.58	0.59

Modified soy protein concentrate 1 (Triple A, Hornsyld, Denmark).

Modified soy protein concentrate 2 (Triple A, Hornsyld, Denmark).

Soybean meal, solvent extracted (Floradale Feed Mill Ltd., Floradale, ON, Canada).

Provided, per kilogram of diet, 12,000 IU vitamin A as retinyl acetate, 1,200 IU vitamin D3 as cholecalciferol, 48 IU vitamin E as dl-α-tocopherol acetate, 3-mg vitamin K as menadione, 18-mg pantothenic acid, 6-mg riboflavin, 600-mg choline, 2.4-mg folic acid, 30-mg niacin, 18-mg thiamin, 1.8-mg pyridoxine, 0.03-mg vitamin B_12_, 0.24-mg biotin, 18-mg Cu from CuSO_4_∙5H_2_O, 120-mg Fe from FeSO_4_, 24-mg Mn from MnSO_4_, 126-mg Zn from ZnSO_4_, 0.36-mg Se from Na_2_SeO_3_, and 0.6-mg I from KI (DSM Nutritional Products Canada Inc., Ayr, ON, Canada).

Calculated based on the NRC (2012) ingredient values with MSPC ingredient values provided by Triple A (Hornsyld, Denmark).

Six barrows (Yorkshire; 22.1 kg BW ± 1.3 kg) were obtained from the University of Guelph Arkell Swine Research Station (Guelph, ON, Canada) and housed individually in a temperature-controlled room (20 to 22 °C) for 7 d prior to surgery. Pigs were surgically fitted with a simple T-cannula at the distal ileum followed by a 1-wk postsurgical recovery period (de Lange et al., 1998). The surgical area was cleaned daily and zinc oxide cream was applied (Crosbie et al., 2020). The experiment was conducted using a replicated 3 × 3 Latin square design with one additional period (*n* = 7 or 6 over four experimental periods). Pigs were weighed at the start of each experimental period to determine individual feed allowance (2.8 × estimated maintenance energy requirements; NRC, 2012). Pigs were fed one of three test diets in two equal meals at 0800 and 1700 h as a wet mash with a water-to-feed ratio of approximately 1:1. After each meal and at 2000 h, 2 liters of additional water was added to the trough in each pen.

Each experimental period lasted for 10 d, with a 7-d adaptation period, followed by 2 d of fecal collection, and subsequently, 2 d of continuous ileal digesta collection for 8 h after the morning meal. For fecal collection, fresh samples were collected twice per day after pen washing and pooled per pig per period. For ileal digesta collection, 10 mL of 10% formic acid was added to a collection bag and then attached to the cannula using an elastic band and was replaced as needed. Digesta was stored in the refrigerator (~4 °C) during collection and pooled per pig per period. Fecal and ileal digesta samples were then stored at −20 °C until analysis.

### Sample Preparation and Chemical Analyses

Prior to analysis, fecal and ileal digesta samples were freeze-dried and finely ground. The particle sizes of test ingredients were determined using a RO-TAP Sieve Shaker (model RX-30 E; W.S. Tyler, Mentor, OH). Test ingredients, experimental diets, fecal, and ileal digesta samples were analyzed for DM (AOAC, 2005; method 930.15). Experimental diets, fecal, and ileal digesta samples were analyzed for titanium content according to Myers et al. (2004) with minor adaptations (digestion for 24 h at 120 °C in 10-mL tubes and addition of H_2_O_2_ after precipitate settled in 100-mL volumetric flasks) and measured using a UV spectrophotometer. The experimental diets and fecal samples were analyzed for gross energy (GE) using a bomb calorimeter (IKA Calorimeter System C 5000; IKA Works Inc., Wilmington, NC) and neutral detergent fiber (NDF) according to Van Soest et al. (1991) using an Ankom 200 Fiber Analyzer (Ankom Technology, Fairport, NY; ingredients and diets only). Test ingredients, experimental diets, and ileal digesta were analyzed for AA using the performic acid oxidized hydrolysis procedure (AOAC, 2005; method 994.12). The AA were quantified via ion-exchange chromatography according to Llames and Fontaine (1994). Test ingredients, experimental diets, and ileal digesta were also analyzed for crude protein (N × 6.25) using a Foss Kjeltech 8200 Auto Distillation Unit (Fisher Scientific, Ottawa, ON) following method 968.06 (AOAC, 2005), and for calcium and phosphorus using inductively coupled plasma mass spectrophotometry (test ingredients and experimental diets only; AOAC, 2005; method 985.01; SGS Canada Inc., Guelph, ON, Canada).

### Calculations and Statistical Analyses

The apparent ileal digestibility (AID) and standard ileal digestibility (SID) of crude protein and AA contents were calculated according to Stein et al. (2007). Basal endogenous losses from Rho et al. (2017) were used to determine the SID of crude protein and AA (i.e., animal genotype and experimental design were identical). The apparent total tract digestibility (ATTD) of an N-free diet with an identical cornstarch:sucrose:oil ratio (Crosbie et al., 2020) as the current test diets was used to determine the ATTD of digestible energy of the MSPC ingredients and SBM using the difference method (Adeola, 2001; Kiarie et al., 2016).

All data were analyzed using the GLIMMIX procedure of SAS (SAS Inst. Inc., Cary, NC). Individual pig was considered the experimental unit. The fixed effects of diet and the random effects of period and diet fed within period were included (e.g., to account for age and physiological state of the pig receiving the diet within a period). When appropriate (*P* < 0.05), differences among individual means were assessed using the Tukey–Kramer post hoc test. A probability of *P* < 0.05 was considered significant, whereas 0.05 < *P* ≤ 0.10 was considered a tendency.

## RESULTS

All pigs remained healthy throughout the study, though the cannulas became dislodged from two of the pigs after period two; an additional period was added to achieve sufficient experimental units for the MSPC1 and MSPC2 treatments. Accuracy of cannula placement in the terminal ileum was confirmed via necropsy at the conclusion of the study.

### Chemical Composition of Ingredients and Experimental Diets

The analyzed chemical composition of MSPC1, MSPC2, and SBM is shown in Table 1. The crude protein content was ~28% greater in the modified soy protein concentrates vs. SBM. Indispensable AA contents were generally within 15% among the protein sources, but Lys was 33% and 38% greater for MSPC1 vs. MSPC2 and SBM, respectively. The calculated and analyzed nutrient contents of the experimental diets were well aligned (Tables 2 and 3).

**Table 3.** Analyzed nutrient composition (as-fed basis) of test diets

Item	MSPC1 diet^1^	MSPC2 diet^2^	SBM diet^3^
Dry matter, %	90.89	90.90	90.92
Crude protein, %	22.21	18.97	22.12
Calcium, %	0.98	0.85	0.75
Phosphorus, %	0.67	0.58	0.54
NDF, %^4^	4.57	5.87	3.64
Gross energy, kcal/kg	3,859	3,977	4,028
Indispensable AA, %			
Arg	1.69	1.50	1.88
His	0.64	0.62	0.80
Ile	1.10	1.13	1.13
Leu	1.90	1.83	1.93
Lys	1.35	1.14	0.92
Met	0.38	0.28	0.69
Phe	1.36	1.36	1.96
Thr	0.92	0.79	0.97
Val	1.09	1.16	1.04
Dispensable AA, %			
Ala	1.15	1.35	0.98
Asp	2.81	2.41	2.08
Cys	0.43	0.22	1.09
Glu	4.36	3.63	3.45
Gly	1.17	1.26	1.34
Pro	1.29	1.32	1.35
Ser	1.85	1.57	1.56
Tyr	0.78	0.79	1.10

Modified soy protein concentrate 1 (Triple A, Hornsyld, Denmark).

Modified soy protein concentrate 2 (Triple A, Hornsyld, Denmark).

Soybean meal, solvent extracted (Floradale Feed Mill Ltd., Floradale, ON, Canada).

NDF = neutral detergent fiber.

### Apparent and Standardized Ileal Digestibilities of Crude Protein and Amino Acids

The AID of crude protein, His, Thr, and Gly was greater for MSPC1 vs. MSPC2 and SBM (*P* < 0.05), which were not different (Table 4). The AID of Arg, Lys, Asp, and Glu was greater for MSPC1 than MSPC2 and SBM (*P* < 0.001), and greater for MSPC2 than SBM (*P* < 0.001). The AID of Ile, Leu, Val, Ala, and Ser was not different between MSPC1 and MSPC2, but was greater for both MSPC1 and MSPC2 vs. SBM (*P* < 0.005). The AID of Met and Cys was greater for MSPC1 and SBM vs. MSPC2 (*P* < 0.001), the AID of Phe was greater for MSPC1 than SBM (*P* < 0.05) and intermediate for MSPC2, and the AID of Pro was greater for MSPC1 than MSPC2 (*P* < 0.05) and intermediate for SBM.

**Table 4.** Apparent ileal digestibility (%) of crude protein and amino acids in two modified soy protein concentrates and soybean meal fed to growing pigs

Item	MSPC1^1^	MSPC2^2^	Soybean meal^3^	SEM^4^	*P*-value
No.^5^	7	7	6		
Crude protein	89.3^a^	85.4^b^	83.6^b^	1.2	0.001
Indispensable AA					
Arg	95.6^a^	93.5^b^	91.4^c^	0.7	<0.001
His	92.0^a^	88.4^b^	89.2^b^	0.9	0.002
Ile	91.0^a^	89.3^a^	85.2^b^	1.3	0.001
Leu	90.9^a^	88.4^a^	84.0^b^	1.2	<0.001
Lys	92.1^a^	87.6^b^	79.0^c^	1.7	<0.001
Met	92.0^a^	85.3^b^	92.8^a^	1.5	<0.001
Phe	91.9^a^	89.9^ab^	88.9^b^	1.0	0.027
Thr	84.8^a^	76.9^b^	76.3^b^	1.9	<0.001
Val	89.1^a^	86.8^a^	80.9^b^	1.7	0.001
Dispensable AA					
Ala	88.2^a^	86.6^a^	74.8^b^	2.9	0.001
Asp	89.6^a^	83.7^b^	74.6^c^	2.0	<0.001
Cys	81.3^a^	59.6^b^	90.1^a^	6.5	<0.001
Glu	93.8^a^	90.1^b^	83.8^c^	1.3	<0.001
Gly	83.9^a^	77.8^b^	77.6^b^	2.4	0.024
Pro	87.8^a^	82.9^b^	83.8^ab^	1.8	0.026
Ser	92.2^a^	86.5^a^	79.9^b^	2.5	0.001

Modified soy protein concentrate 1 (Triple A, Hornsyld, Denmark).

Modified soy protein concentrate 2 (Triple A, Hornsyld, Denmark).

Soybean meal, solvent extracted (Floradale Feed Mill Ltd., Floradale, ON, Canada).

Maximum value of standard error of the means.

Number of experimental units.

The SID of crude protein was greater for MSPC1 than SBM (*P* < 0.005), whereas MSPC2 was intermediate (Table 5). The SID of Arg, Asp, and Glu was greater for MSPC1 than MSPC2 and SBM (*P* < 0.001), and greater for MSPC2 than SBM (*P* < 0.001). The SID of His, Thr, Gly, and Pro was greater for MSPC1 than either MSPC2 or SBM (*P* < 0.05), which were not different. The SID of Ile, Leu, Lys, Phe, Val, Ala, and Ser was greater for both MSPC1 and MSPC2 vs. SBM (*P* < 0.005) and the SID of Met and Cys was greater for both MSPC1 and SBM vs. MSPC2 (*P* < 0.005).

**Table 5.** Standardized ileal digestibility (%) of crude protein and amino acids in two modified soy protein concentrates and soybean meal fed to growing pigs

Item	MSPC1^1^	MSPC2^2^	Soybean meal^3^	SEM^4^	*P*-value
No.^5^	7	7	6		
Crude protein	96.9^a^	94.3^ab^	91.3^b^	1.2	0.001
Indispensable AA					
Arg	99.0^a^	97.3^b^	94.4^c^	0.7	<0.001
His	96.1^a^	92.7^b^	92.5^b^	0.9	0.001
Ile	94.7^a^	92.9^a^	88.8^b^	1.3	0.001
Leu	94.7^a^	92.4^a^	87.8^b^	1.2	<0.001
Lys	95.8^a^	92.0^a^	84.5^b^	1.7	<0.001
Met	95.5^a^	90.1^b^	94.7^a^	1.5	0.003
Phe	97.7^a^	95.7^a^	92.9^b^	1.0	0.001
Thr	93.1^a^	86.5^b^	84.2^b^	1.9	0.001
Val	94.4^a^	91.9^a^	86.5^b^	1.7	0.001
Dispensable AA					
Ala	94.0^a^	91.6^a^	81.7^b^	2.9	0.002
Asp	93.2^a^	87.9^b^	79.5^c^	2.0	<0.001
Cys	87.4^a^	65.1^b^	92.5^a^	6.5	0.001
Glu	96.6^a^	93.3^b^	87.2^c^	1.3	<0.001
Gly	99.1^a^	91.8^b^	90.8^b^	2.4	0.006
Pro	109.5^a^	104.2^b^	104.7^b^	1.8	0.013
Ser	96.2^a^	91.3^a^	84.6^b^	2.5	0.001

Modified soy protein concentrate 1 (Triple A, Hornsyld, Denmark).

Modified soy protein concentrate 2 (Triple A, Hornsyld, Denmark).

Soybean meal, solvent extracted (Floradale Feed Mill Ltd., Floradale, ON, Canada).

Maximum value of standard error of the means.

Number of experimental units.

### Apparent Total Tract Digestibility of Energy

The ATTD of GE (%) was greater for MSPC1 than MSPC2 (*P* < 0.01) and intermediate for SBM (Table 6). The DE (kcal/kg) was greater for MSPC1 than either MSPC2 or SBM (*P* < 0.005), which were not different.

**Table 6.** Apparent total tract digestibility (ATTD, %) of gross energy (GE) in two modified soy protein concentrates and soybean meal fed to growing pigs

Item	MSPC1^1^	MSPC2^2^	Soybean meal^3^	SEM^4^	*P*-value
No.^5^	7	7	6		
ATTD, %					
GE	90.4^a^	73.6^b^	83.9^ab^	4.7	0.005
DE, kcal/kg^6^	4,677^a^	3,755^b^	4,037^b^	239	0.003

Modified soy protein concentrate 1 (Triple A, Hornsyld, Denmark).

Modified soy protein concentrate 2 (Triple A, Hornsyld, Denmark).

Soybean meal, solvent extracted (Floradale Feed Mill Ltd., Floradale, ON, Canada).

Maximum value of standard error of the means.

Number of experimental units.

DE = digestible energy.

## DISCUSSION

The objective of the current study was to determine the SID of crude protein and AA and digestible energy of two MSPC and conventional SBM (dehulled, solvent extracted) fed to growing pigs, of which both MSPC1 and MSPC2 were digestible sources of AA. However, MSPC1 had 33% and 38% greater total Lys content vs. MSPC2 and SBM, respectively, which when combined with the SID coefficients, resulted in 35% and 46% greater SID Lys content (42.8 vs. 27.7 and 23.2 g SID Lys/kg product for MSPC1, MSPC2, and SBM, respectively). Moreover, the MSPC1 had 7% greater GE than SBM and greater ATTD of GE vs. MSPC2, resulting in greater DE values for MSPC1 vs. either MSPC2 or SBM, despite relatively greater NDF contents. The improvements in SID Lys and DE for MSPC1 could be partially mediated by relatively smaller, though more variable, particle size vs. MSPC2 and SBM (Table 1). Therefore, MSPC1 is a digestible protein source for growing pigs that also provides more DE than conventional SBM.

In the current study, the crude protein and Lys contents and the SID coefficients for SBM closely reflect the values determined in previous studies (e.g., NRC, 2012; Navarro et al., 2018) when considering typical variation among SBM sources and methods for determining SID of amino acids (van Kempen et al., 2002; Messad et al., 2016). Therefore, the source of SBM used in the current study can be considered reflective of current SBM products and an appropriate comparison for the MSPC products. Previous research demonstrated a 35% reduction in trypsin inhibitor activity for a MSPC product vs. SBM (Kiarie et al., 2021), which supports the improvements for crude protein and AA SID coefficients for MSPC1 vs. SBM found in the current study. Others have noted that further processing of soybean products increased crude protein contents, but may also reduce the digestibility of certain amino acids. For example, Navarro et al. (2017) demonstrated reduced SID of Lys for weanling pigs when soybean meal was treated with difference combinations of enzymes, despite improvements in crude protein contents (vs. conventional, dehulled soybean meal). The specific reductions in SID of Lys vs. other AA during certain processing methods indicate that heat damage occurred (González-Vega et al., 2011). Indeed, in the current study, the Lys:crude protein ratio was 4.80% for MSPC2 vs. 6.47% and 5.83% for MSPC1 and SBM, respectively, signifying greater extent of heat damage for MSPC2, which was even greater than the heat damage that occurred for other processed soybean products (e.g., fermented soybean meal, enzyme-treated soybean meal, extruded soybean meal, and soy protein concentrate; Rojas and Stein, 2013; Cotten et al., 2016; Navarro et al., 2017). Therefore, further processing of soybean products can improve AA digestibility for growing pigs, but manufacturers must ensure that overheating does not occur as part of the quality control procedures.

Highly digestible protein ingredients are often included in pig diets for the first phase(s) after weaning to attenuate the postweaning growth lag caused by exposure to novel pathogens and food allergens, an immature gastrointestinal tract, and other environmental stressors that reduce feed intake (Campbell and Dunkin, 1983; Whang et al., 2000; Lallès et al., 2004). Based on the results of the current study, MSPC could also be a suitable protein source for newly weaned pigs, due to high AA digestibility and DE contents, and possibly, reduced antinutritional factors compared to soybean meal (Kiarie et al., 2021). However, it is noted that the digestibility coefficients are typically greater for growing vs. newly weaned pigs (Sauer et al., 2012), and that nursery diets are more likely to include soybean products that have undergone further processing to remove or deactivate various antinutritional factors vs. other swine production stages.

The use of MSPC to partially replace SBM in broiler chick diets improved ADG and feed efficiency during the first week after hatch and reduced plasma uric acid concentration and improved breast yield at 42 d of age, which indicates an improvement in postabsorptive utilization of AA for net protein gain (Kiarie et al., 2021). Better utilization of dietary AA for protein gain in sellable tissues could have been due to a more optimal AA balance and/or the removal of antinutritional factors that activate the immune system and cause damage to the gastrointestinal tract (e.g., glycinin, β-conglycinin, lectin; Kiarie et al., 2021). For example, glycinin and β-conglycinin have been shown to increase the proliferative and apoptotic indexes in the duodenum of weaned pigs (Zhao et al., 2010), which partition AA and energy towards repair of the gastrointestinal tract vs. growth of salable tissues (i.e., muscle). It is possible that nursery pigs fed MSPC to partially replace SBM would also exhibit improvements in growth performance (ADG and feed efficiency) similar to broiler chicks, but this has yet to be confirmed experimentally.

In summary, further processing of soybean products to generate MSPC generally improved the SID of crude protein and AA and DE contents. However, for some products, overheating may have occurred during processing, specifically reducing the SID Lys content. Therefore, the MSPC1 was a better source of SID Lys and DE than either MSPC2 or SBM and could be used as a highly digestible protein ingredient in swine rations.
